# Optimization Method for Energy Saving of Rural Architectures in Hot Summer and Cold Winter Areas Based on Artificial Neural Network

**DOI:** 10.1155/2022/2232425

**Published:** 2022-03-02

**Authors:** Yong Yang, Xiancheng Liu, Congxiang Tian

**Affiliations:** ^1^School of Urban Construction, Yangtze University, Jingzhou 434100, China; ^2^Yangtze University College of Arts and Sciences, Jingzhou 434100, China

## Abstract

With the phased spatial planning of the rural revitalization strategy, the proportion of architecture energy consumption in the overall social energy consumption is also increasing year by year. Considering the hot summer and cold winter areas, the proportion of architecture energy consumption in the total energy consumption is very large. The ecological environment and natural resources have been greatly threatened, and the issue of energy conservation and environmental protection is imminent. Energy consumption prediction and analysis is an important branch of building energy conservation in the field of building technology and science. Aiming at the energy consumption characteristics of rural architectures in areas with hot summer and cold winter, this paper proposes a method for constructing a neural network model. When building a neural network, the dataset is called and the function is applied randomly to training samples. The data are used for simulation tests to analyze the fit between the predicted results and the calculated results. Flexible forecasting of specific target building energy consumption is achieved, which can provide optimization strategies for updating and adjusting architecture energy efficiency design. The experimental analysis benchmark parameters and the output value in the dataset are compared with the target simulation value. The relative error is less than 4%, and the average relative error value (mean) and the root mean square error (RMSE) value are both controlled within 2%. It is proved that the method in this paper can directly reflect the evaluation of energy consumption by the neural network and realize the high-speed conversion of the generalized model to the concrete goal, which has a certain value and research significance.

## 1. Introduction

With the development of our country's economy and the continuous improvement of people's living standards, people's requirements for indoor comfort are also getting higher and higher. In China, the region with hot summer and cold winter generally refers to the middle and lower reaches of the Yangtze River and its surrounding areas. Most of the regions have extremely strong geographical and climatic characteristics, that is, sultry summer, wet and cold winter, small diurnal temperature range, large annual precipitation, and less sunshine. In some areas with hot summer and cold winter, the central heating in the north is not used in winter, the indoor thermal environment is relatively poor, and the heating demand will continue to increase. In this way, the cooling and heating loads of architectures in summer and winter will increase accordingly, which is not conducive to building energy conservation [1].

The team of Japanese scholar Yoshiyuki Shimoda analyzed and explored the characteristics of architecture energy consumption from the aspect of architecture equipment. Relying on the theoretical system and application principles of energy management, a lot of research work and simulation analysis have been carried out. The main influencing factors and their weights of architecture energy consumption are discussed from different perspectives, and some scientific research results have been obtained [2]. Based on the perspective of thermal engineering, Marty et al. conducted a comprehensive analysis of fuel dynamic factors such as architecture area, envelope thermal performance, and heating and cooling equipment systems that affect energy consumption during architecture operation and predicted the changing trend of architecture energy consumption in the short term of the future [3]. The research results show that in addition to being limited by most climatic conditions and the current status of existing building renovations, the energy consumption load generated by the HVAC system operating under normal operating conditions will fluctuate accordingly due to the dynamic changes in the population composition of users for the building space [4]. Balaban and Puppim de Oliveira used the eQUEST dynamic energy simulation software to establish an architecture energy consumption model and set up orthogonal experiments, which include window-to-wall ratio, window-to-ground ratio, and other constraints. Moreover, they studied the influencing factors of high-rise architecture energy consumption [5]. The model can not only quantitatively analyze the influencing factors of architecture energy consumption changes but also determine the directionality between the influencing factors of building energy consumption and obtain the quantitative relationship between a single factor variable and the total architecture energy consumption under the control of factor interaction [6].

When the energy consumption factors and prediction models of the above research architectures are comprehensively analyzed, it is necessary to coordinate the traditional factors that affect the building energy consumption, such as the thermal performance of the envelope structure, and combine the external environmental factors that affect the energy consumption changes outside the architecture shape. However, due to the unfavorable effects of fuzzy boundary conditions, local extreme values of functions, weakly correlated interference terms, etc., the research points have limitations, and the energy consumption model is relatively targeted [7]. From the research status at home and abroad, it can be seen that in recent decades, foreign developed countries have increasingly stricter requirements on the energy-saving rate of buildings and thermal engineering of architecture envelopes. There have also been fruitful achievements for the related research on the enclosure structure of rural architectures in hot summer and cold winter areas. However, most of these studies did not analyze the energy-saving standards, nor did they discuss the cost-input relationship of the architecture envelope from the perspective of the owner [8]. In order to avoid the high energy consumption of qualified buildings, it is necessary to limit the annual energy consumption index of buildings in the design process, supplemented by operational calculation means, which is convenient for owners, designers, engineers, construction parties, and other parties to implement. Only by controlling the energy consumption in the design process can the actual energy consumption in the building operation stage be reduced.

The Nash–Sutcliffe efficiency coefficient (the closer it is to 1, the better the prediction effect is) predicted by the ANN (artificial neural network) for building cold resistance, heat resistance, and insulation energy consumption is 0.994, 0.993, and 0.996, respectively. In order to solve the trade-off between thermal comfort and energy consumption of residential buildings, ANN and multiobjective genetic algorithm (NSGA-II) were combined to take the parameters related to envelope structure as input. The thermal comfort and energy consumption are taken as output. The results show that the final prediction errors of thermal comfort and energy consumption are less than 4% and 1%, respectively.

Taking three rural architectures in hot summer and cold winter areas as examples, this paper uses the drawing information of the original construction drawings that have passed the energy-saving review and applies the learning simulation performance of BP neural network to construct energy consumption models for rural architectures in hot summer and cold winter areas. Combined with the national energy-saving standards, the influence of the thermal performance of rural architecture envelope on the building's annual energy consumption and the relationship between the initial investment in thermal insulation of the envelope and its service life are discussed. It can provide a certain reference for the thermal design of the envelope structure of the public architecture in the hot summer and cold winter area and also provide economic advice for the owner to choose the envelope structure, which can improve the owner's enthusiasm for architecture energy conservation.

The main innovations of this paper are as follows:

1. Considering the subjective and objective factors that affect the energy consumption of rural architectures, the three most representative influencing factors of architecture energy consumption are selected for modeling.
2. Multiple linear regression analysis and artificial neural networks are introduced to build a public architecture energy consumption model suitable for areas with hot summer and cold winter from a mesoscopic perspective.

## 2. Related Work

### 2.1. Climatic Characteristics of Hot Summer and Cold Winter Region

The GB 50176-2016 code for Thermal Engineering Design of Civil Buildings divides China's climate into five regions: very cold, cold, hot in summer and cold in winter, hot in summer and warm in winter, and mild in winter. The hot in summer and cold in winter region mainly refers to the middle and lower reaches of the Yangtze River, which is in the east of the Sichuan Basin, north of the Nanling, and south of Longhai Line. The climate is characterized by sultry summer (the temperature in July is about 5°C higher than that of other regions at the same latitude in the world). Winter is cold and bleak (the temperature in January is 8–10°C lower than that of other parts of the world at the same latitude). The annual relative humidity is as high as 80%. Therefore, the summer load of buildings in this area is mainly refrigeration, and the winter load is mainly heating.

### 2.2. Characteristics of Rural Architectures

**Figure 1:**
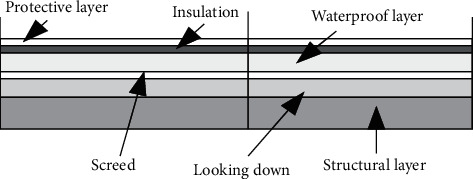
Inverted roof.

1. The roof is the architecture envelope with the longest exposure to solar radiation. The inverted roof is a roof practice in which the order of the thermal insulation layer and the waterproof layer in the traditional roof is exchanged, and the thermal insulation layer is placed on the waterproof layer. The inverted roofing method stipulated in “Technical Regulations for Inverted Roof Engineering” (JGJ230-2010) is shown in Figure 1 [9].
2. Placing the thermal insulation layer on top of the waterproof layer can effectively protect the waterproof layer, reduce the direct damage to the waterproof layer in the harsh outdoor environment, and prolong the life of the waterproof layer. The inverted roof method is similar to the traditional roofing method. It does not add additional construction difficulty, and the total cost does not increase much. However, it can reduce the repair cost of the waterproof layer, prolong the service life of the roof, and bring long-term benefits [10].
3. However, several problems have also emerged in the practical application of inverted roofs, such as the infiltration of rainwater into the gaps between the roof insulation boards, which can result in a decrease in the overall insulation performance of the roof. The cracking of the structural layer directly damages the waterproof layer, and once the waterproof layer is damaged, the repair cost is greater. The waterproof layer is hidden below, and it is difficult to accurately locate the water seepage point. Due to the protective effect of waterproof materials on thermal insulation materials, some unscrupulous enterprises use low-cost and inferior waterproof materials, which will leave hidden dangers to safety.
4. Inverted roofs offer several advantages over traditional roofs. However, in practical application, it is necessary to consider the specific situation of the project and choose the roof construction method reasonably.
5. The thermal insulation technology of the external wall of the architecture mainly includes external thermal insulation of the external wall, internal thermal insulation of the external wall, composite thermal insulation of the external wall inside and outside, and self-thermal insulation of the external wall. The determination of the thermal insulation form should comprehensively consider the influence of factors such as the climate where the architecture is located, the specific type and structural form of the building, and the economic benefits in the entire life cycle. Next, these four common exterior wall insulation techniques will be discussed [12].

### 2.3. Technical Process of Thermal Insulation Engineering

**Figure 2:**
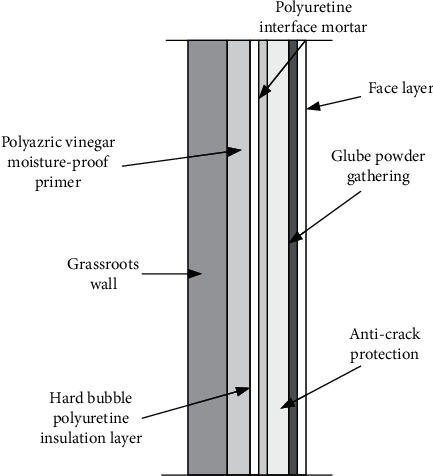
External thermal insulation system for external walls.

JGJ144-2005 Technical Specification for External Wall Thermal Insulation Engineering stipulates that the external wall thermal insulation system is composed of thermal insulation layer, plastering layer, fixing material (adhesive, anchoring agent, etc.), and coating layer, and fixed on the external surface of the external wall, which is called “external thermal insulation system” [13]. The outer insulation layer adopts the method of “rigid foam polyurethane,” as shown in Figure 2.

The advantages of an external thermal insulation system for external walls are that the thermal insulation layer is between the outside and the wall, which can reduce the stress effect of temperature changes on the wall and avoid the damage and corrosion of the base wall caused by external carbon dioxide, water, harmful gases, ultraviolet rays, and other factors. The overall thermal insulation effect of the external thermal insulation system is good, which is beneficial to improve the indoor environmental quality and the operation of the HVAC system. Under the same thermal insulation effect, the thickness of the thermal insulation material of the external thermal insulation system is thinner, and the change of direction reduces the total consumption of thermal insulation materials and increases the useable area in the room. It effectively eliminates the thermal bridge phenomenon, reduces the indoor heat load of the architecture, and is beneficial to building energy conservation [14].

After simulating and analyzing the relationship between the annual energy consumption of three public architectures with different shapes and the thermal work of the envelope structure, it is found that the strengthening of the envelope structure can reduce the cooling demand in summer and the heating demand in winter, which is beneficial to the energy saving of the architecture throughout the year. For super-high-rise hotel architectures with a high window-to-wall ratio (Architecture A), thermal enhancement of external window thermal work can reduce the building's heating and cooling load by up to about 40%. The energy-saving effect is far better than strengthening external wall and roof thermal work [15]. Comparing two low-rise rural architectures with similar shapes, it is found that the energy-saving potential of the architecture (Architecture C) with the 2005-year energy-saving standard is significantly greater than that of the architecture (Architecture B) with the 2015-year energy-saving standard [16].

## 3. BP Neural Network

### 3.1. Design of Neural Network

The BP neural network can have one or more hidden layers. However, some studies have shown that the BP neural network with a single hidden layer can continuously approximate any continuous function, and the three-layer BP neural network can map an n-dimensional space. There is a functional relationship between building benchmark parameters and architecture cooling and heating energy consumption. Therefore, in this paper, the number of layers of the BP neural network is determined to be three layers, and the hidden layer is set to one layer. In the single-hidden layer, the number of neurons is often determined according to the number of input and output neurons [15]. Because of the large number of neurons in the input layer, according to empirical formula (1), the number of neurons in the hidden layer can be tentatively set to 15. To check the operation of the network model in the future, 15 neurons may not necessarily make the network model achieve the best effect. To avoid the phenomenon of overfitting or the inaccuracy of the network model, it is necessary to adjust the number of neurons in the hidden layer, perform repeated training of the neural network, and then select the optimal number of neurons according to the training results to determine the number of neurons in the hidden layer [17].

The empirical formula for the number of hidden layer nodes of the neural network model in this paper is

(1) $$\begin{array}{c} h=\sqrt{m+n}+a, \end{array}$$

where *h* is the number of hidden layer nodes; *m* and *n* are the number of the input layer and output layer nodes, respectively; and *a* is an adjustment constant between 1 and 10.

In view of the large number of neuron nodes in the input layer, according to empirical formula (1), the number of neurons in the hidden layer can be temporarily set as 15, which is to be tested later in the network model operation, that is, 15 neurons cannot necessarily make the network model achieve the best effect [18]. In order to avoid the overfitting phenomenon or the misalignment of the network model, it is necessary to adjust the number of neurons in the hidden layer and conduct repeated training of the neural network. Then, select the optimal number of neurons according to the training results so as to determine the number of neurons in the hidden layer.

### 3.2. Implementation of Neural Network Model

#### 3.2.1. Data Calling and Processing

To establish an accurate energy consumption model, when building a neural network, 800 sets of datasets in Excel are called, and the rand() function is used to randomly select 770 sets of training samples. The remaining 30 sets of data are used for simulation testing to analyze the agreement between the results and the calculated results. The load command is used to call the influencing factors and energy consumption values in the basic data of architecture energy consumption, and they are named as variables “*p*” and “*t*,” respectively.

#### 3.2.2. Normalization of Data

In neural network learning, it is necessary to normalize the sample data, that is, the input data of the network and the corresponding expected output value. They are temporarily normalized to a predetermined interval, and after the network learns and trains, the previously normalized values must be converted into actual values[19]. In MATLAB software, the map-min-max function is used to normalize the data to [0, 1]. The normalized linear transformation algorithm is expressed as

(2) $$\begin{array}{c} y=\frac{x-x_{\min}}{x_{\max}-x_{\min}}, \end{array}$$

where *x* is the input variable; *x*_min_ is the minimum value of the input variable *x*; *x*_max_ is the maximum value of the input variable *x*; and *y* is the output variable after normalization.

#### 3.2.3. Parameter Attribute Setting of Neural Network

**Figure 3:**
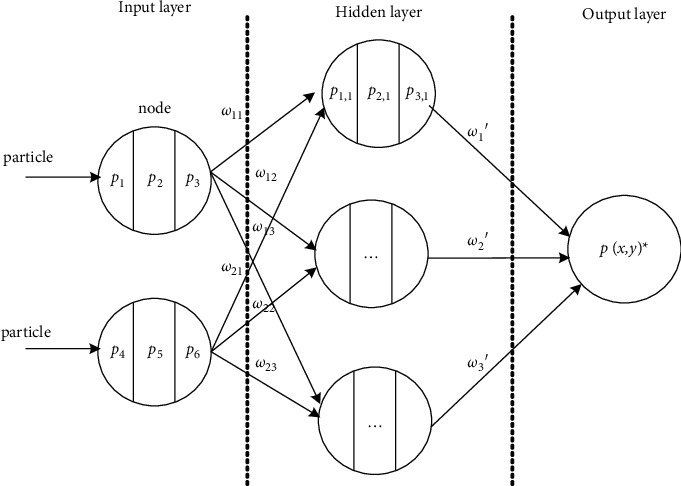
Neural network structure.

The network is created, and the network properties are set. According to the initial values of the data designed in the previous section, combined with empirical formula (1), the number of neurons in the hidden layer is 15, and the newff function is used to create the network. In this paper, the Levenberg–Marquardt algorithm (namely, “trainlm”) is selected as the training function of the network because it has the characteristics of the largest memory requirement, the fastest convergence speed, and no local extremum. The neural network structure is shown in Figure 3.

After creating the network, the network properties are set. It mainly includes that the number of iterations epochs for network training is set to 2000 times, the training required accuracy goal is set to 1*e* − 3, and the learning rate *lr* is set to 0.01.

The setting of network parameters is flexible and controllable according to the different research objects and the size of the sample dataset, and the network attribute module is set twice. The results of the model are recorded and analyzed after several rounds of operations. The network structure with the smallest error of the weight function is selected after multiple corrections, and it is reset to the parameter properties of the algorithm model.

#### 3.2.4. Training of Neural Network

**Figure 4:**
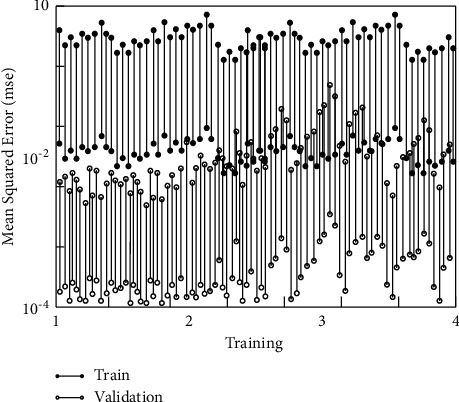
Neural network performance verification.

After setting the parameters, the neural network is trained. The useful method is [net, tr]=train(net, pl, tl), the network reaches the best state through 4 repeated learning and training processes, the iterative algorithm converges very fast, the accuracy value is 0.000998, and the training is completed. The error reduction process is shown in Figure 4.

From the display of the distribution of the test results, the data have a high degree of agreement, the network model structure is stable, and there is no gradient explosion and local extremum problems.

#### 3.2.5. Denormalization of Data

After the neural network reading is completed, the map-min-max function is used to denormalize the results. The parameters of the output layer with a threshold of [0, 1] are restored to the corresponding annual energy consumption values for cooling and heating, and the real value of energy consumption is recorded. So far, the training, simulation testing, and denormalization of the network model are completed. The detailed algorithm is as follows:

  net = train (net, p_train, t_train);
  t_sim = sim (net, p_test);
  T_sim = mapminmax (“:reverse,” t_sim, ps_output).

## 4. Experimental Analysis

### 4.1. Energy-Saving Information of Simulated Architectures

Take three rural architectures with different shapes in the hot summer and cold winter area as an example. Combined with the drawing information of the construction drawings that have passed the energy-saving review, an energy-saving model is built in the simulation software BECS to analyze the impact of thermal performance on the building's annual energy consumption. Architecture A and Architecture B are used to discuss the influence of the window-to-wall ratio on architecture energy consumption under the same energy-saving standard. The impact of the 2005 and 2015 standards on architecture energy consumption is specifically discussed using Architecture B and Architecture C. The extracts from the energy-saving report are shown in Table 1.

GB50189-2005 proposed a 50% energy-saving target, that is, compared with the public architectures (“benchmark architectures”) built in the 1980s of the early days of Reform and Opening Up, the energy-saving rate should reach 50%. The energy-saving rate of the “reference architecture” in the software corresponds to 50%, and GB50189-2015 increases its value to 65%.

The completion time of the construction drawings of Architectures A and B is after the implementation of the GB50189-2015 standard, and the energy-saving rate is 67.7% and 67.4%, which can meet the requirements of the 15-year standard. The construction drawing of Architecture C is completed before the implementation of the GB50189-2015 standard, and the energy-saving rate is 62.4%, which can meet the 05-year standard, but cannot meet the 15-year standard.

According to the working principle of the simulation software, the reference architectures are designed according to the 65% benchmark, that is, the energy-saving rate of the software output reference architectures is 65%. In the simulation results, Architecture C (energy-saving rate of 62.4%) does not meet the energy-saving requirements because it is calculated according to the 65% energy-saving rate of GB50189-2015. However, the completion time of architecture construction drawings is before the implementation of GB50189-2015, and the energy-saving rate requirement is designed according to 50% of GB50189-2005, that is, Architecture C meets the energy-saving requirements at that time. The energy-saving rate mentioned in the 05-year standard is only to illustrate that Architecture C is in line with the energy-saving requirements at that time. To reflect the comparability of different architectures under the same conditions, the 65% energy-saving rate of GB50189-2015 will be adopted as the reference architecture in the follow-up.

**Figure 5:**
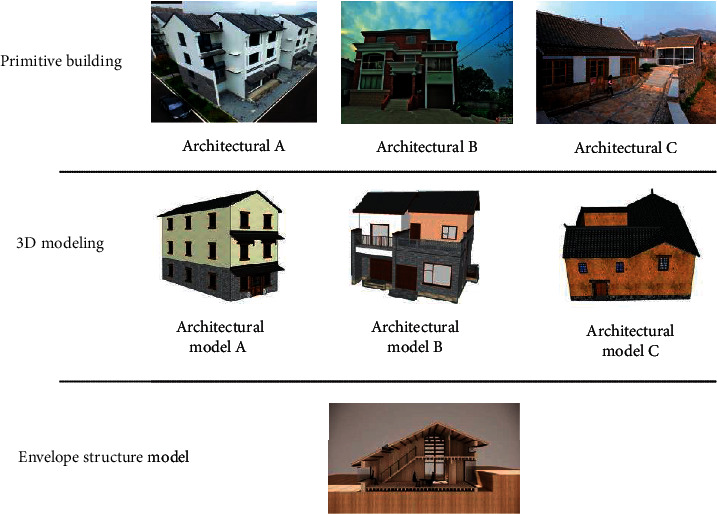
Structure and model of architectures.

For external wall insulation materials, the materials selected for Architectures A and B have better thermal insulation performance (rock wool tape and rock wool board), and the thickness is thicker. The material selected for Architecture C has relatively poor thermal insulation performance (micro-bead inorganic thermal insulation mortar), and the thickness is thinner. The roof insulation materials for the three architectures are the same, but the thickness of Architecture C is the lowest. Architectures A and B also have better thermal insulation than Architecture C. Overall, the overall thermal performance of the envelope structure of Architectures A and B is better than that of Architecture C. The architecture structure and model are shown in Figure 5.

### 4.2. Influence of Roof Insulation on Energy Consumption

**Figure 6:**
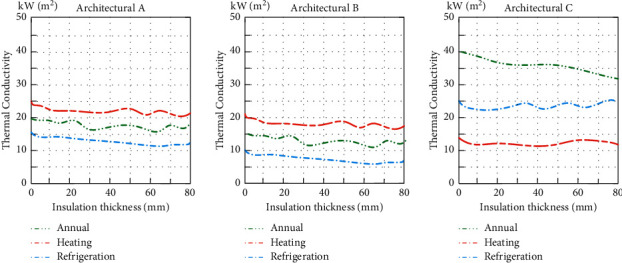
Relationship between the thickness of the thermal insulation material and the annual energy consumption of the roof.

The actual orientation, room function, and envelope structure of Architectures A, B, and C are selected to discuss the influence of roof insulation thickness on architecture energy consumption. When the thermal insulation material of the roof changes, the relationship between the annual energy consumption of the roof of Architectures A, B, and C is shown in Figure 6.

The simulation results show that the thickness change of the roof insulation material has little effect on the annual energy consumption of Architectures A and B but has a greater impact on the heating energy consumption of Architecture C. The roof of Architecture C adopts the same insulation material as A and B, and the overall heat transfer parameters with the same roof insulation thickness are between A and B. Observing the specific structure of Architecture C, it can be found that the roof of Architecture C accounts for a higher proportion of the total envelope structure, so its roof insulation has a more significant impact on the annual energy consumption.

### 4.3. Influence of External Wall Insulation on Energy Consumption

**Figure 7:**
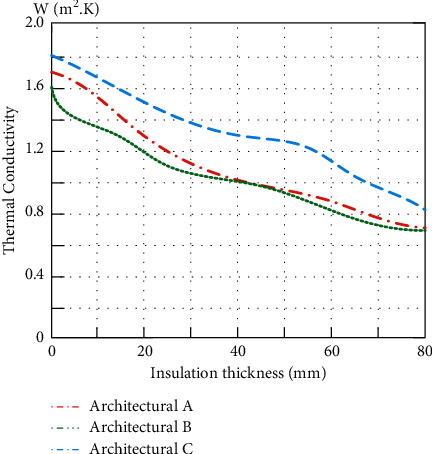
Relationship between the thickness of external wall insulation material and heat transfer coefficient.

**Figure 8:**
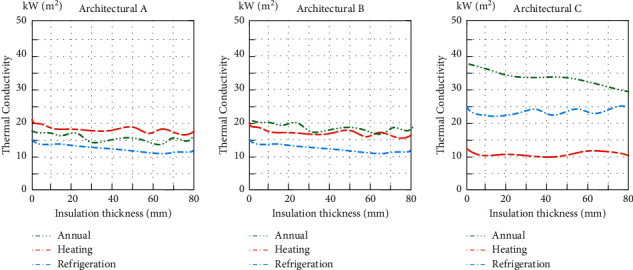
Relationship between thermal insulation material thickness and annual energy consumption of external walls.

Except for external wall insulation, other parameters are kept consistent with the actual situation. When the insulation material of the external wall changes, the relationship between the heat transfer coefficient and the annual energy consumption of the external walls for the three architectures is shown in Figures 7 and 8.

As can be seen from Figure 7, Architecture C refers to the 05-year energy-saving standard and selects the thermal insulation material (micro-bead inorganic thermal insulation mortar) with poor thermal performance. Architectures A and B refer to the 15-year standard and select insulation materials (rock wool) with better thermal performance. Therefore, under the same thickness of thermal insulation materials, the thermal performance of the external walls of Architectures A and B is better than that of Architecture C.

From the simulation results in Figure 8, it can be seen that the increase of the thermal insulation thickness of the external wall can significantly reduce the annual heating energy consumption (dotted line) of the architecture and has a relatively small impact on the annual cooling energy consumption (stipple line) of the architecture, and the combined effect is conducive to reducing the annual energy consumption (solid line) of the architecture.

## 5. Conclusion

This paper mainly studies the energy-saving optimization design of rural buildings in hot summer and cold winter areas. The main work is as follows:

1. The approximate function of each variable with the energy consumption value drawn by the function graph is used to fit the regression curve.
2. By analyzing the influence of various factors on the energy consumption of rural buildings, combined with the actual situation of the project, the energy-saving design values of the benchmark parameters of rural architectures are obtained with high efficiency and high precision, which can provide a reference for energy-saving optimization strategies.
3. The experimental results show that the neural network does have the advantage of high-precision computing for building energy consumption It is verified that the energy consumption model of rural buildings in hot summer and cold winter zone based on artificial neural network is scientific.

Through experiments, the energy-saving design characteristics of various buildings are verified. It is also proved that the research content of this paper will seriously affect the reduction of annual energy consumption value (11.79∼39.12%) on the characteristics of exterior windows of buildings. It is higher than the traditional building reduction (9.03∼12.95%). The overall increase is 8.50%.

Compared with professional energy consumption simulation software, the energy consumption prediction of neural network is modeled from the input and output law of data, without considering the heat conduction process between the building itself and the environment and the calculation principle of cold and heat load of the building. The simulation accuracy also has room for improvement. If the research problem has a large amount of computation and the accuracy requirement is relatively low, the neural network model shows excellent computational performance. However, for problems requiring high accuracy, it is recommended to use professional energy consumption simulation software. The results will be more accurate.

## Figures and Tables

**Table 1:** Energy-saving information of simulated architectures.

Classification		Architecture A	Architecture B	Architecture C
Features		Township house	Township house	Township house
Layers		3	2	1
Height		15.10 m	10.30 m	5.00 m
Above ground area		258 m^2^	196 m^2^	98 m^2^
Compass direction		225.7°	74.8°	0°
Factor		0.12	0.15	0.26
Roof	Insulation materials	Extruded polystyrene board	Extruded polystyrene board	Extruded polystyrene board
	Insulation thickness	70 mm	60 mm	55 mm
	Heat transfer coefficient	0.45 W/(m^2^•K)	0.34 W/(m^2^•K)	0.48 W/(m^2•^K)
External wall	Insulation materials	Rock wool tape	Rock wool board	Micro-bead inorganic thermal insulation mortar
	Thermal conductivity	0.048 W/(m•K)	0.040 W/(m•K)	0.070 W/(m•K)

## Data Availability

The data used to support the findings of this study are available from the corresponding author upon request.
